# Circulating Reactive Oxygen Species in Adults with Congenital Heart Disease

**DOI:** 10.3390/antiox11122369

**Published:** 2022-11-30

**Authors:** Inne Vanreusel, Dorien Vermeulen, Inge Goovaerts, Tibor Stoop, Bert Ectors, Jacky Cornelis, Wendy Hens, Erwin de Bliek, Hilde Heuten, Emeline M. Van Craenenbroeck, An Van Berendoncks, Vincent F. M. Segers, Jacob J. Briedé

**Affiliations:** 1 Department of Cardiology, Antwerp University Hospital, 2650 Edegem, Belgium; 2 Research Group Cardiovascular Diseases, GENCOR, University of Antwerp, 2000 Antwerp, Belgium; 3 Cardiac Rehabilitation Centre, Antwerp University Hospital, 2650 Edegem, Belgium; 4 Department of Rehabilitation Sciences and Physiotherapy, Faculty of Medicine and Health Sciences, MOVANT Research Group, University of Antwerp, 2000 Antwerp, Belgium; 5 Department of Toxicogenomics, School of Oncology and Developmental Biology (GROW), Maastricht University, 6211 MD Maastricht, The Netherlands

**Keywords:** oxidative stress, reactive oxygen species (ROS), superoxide anion radical, inflammation, exercise capacity, endothelial (dys)function, “heart defects, congenital” [Mesh]

## Abstract

Oxidative stress is an important pathophysiological mechanism in the development of numerous cardiovascular disorders, but few studies have examined the levels of oxidative stress in adults with congenital heart disease (CHD). The objective of this study was to investigate oxidative stress levels in adults with CHD and the association with inflammation, exercise capacity and endothelial function. To this end, 36 adults with different types of CHD and 36 age- and gender-matched healthy controls were enrolled. Blood cell counts, hs-CRP, NT-proBNP, fasting glucose, cholesterol levels, iron saturation and folic acid concentrations were determined in venous blood samples. Levels of superoxide anion radical in whole blood were determined using electron paramagnetic resonance spectroscopy in combination with the spin probe CMH. Physical activity was assessed with the IPAQ-SF questionnaire. Vascular function assessment (EndoPAT) and cardiopulmonary exercise testing were performed in the patient group. Superoxide anion radical levels were not statistically significantly different between adults with CHD and the matched controls. Moreover, oxidative stress did not correlate with inflammation, or with endothelial function or cardiorespiratory fitness in CHD; however, a significant negative correlation with iron saturation was observed. Overall, whole blood superoxide anion radical levels in adults with CHD were not elevated, but iron levels seem to play a more important role in oxidative stress mechanisms in CHD than in healthy controls. More research will be needed to improve our understanding of the underlying pathophysiology of CHD.

## 1. Introduction

Congenital heart diseases (CHDs) comprise a wide range of cardiac malformations [1]. Medical and surgical advances have dramatically increased the survival of patients with CHD, leading to a continuously growing number of children, adolescents and adults with CHD [2,3]. Nevertheless, CHD patients have lower physical fitness [3,4,5], reduced quality-of-life [6] and worse prognosis [7,8] compared to healthy individuals of similar age. The development and progression of heart failure (HF) is the main cause of morbidity and mortality in this population [9,10]. Patients with HF induced by CHD and patients with HF induced by other etiologies share many characteristics, including exercise intolerance [4,11,12,13], ventricular dysfunction [1,14,15], increased inflammatory cytokine levels [16] and neurohormonal activation [17]. To improve therapy and preventive strategies, we need a better understanding of the underlying pathophysiological mechanisms.

In patients with HF induced by other etiologies, increased oxidative stress is implicated in the pathogenesis of cardiac injury and the disease progression [18,19]. Under physiological conditions, the most common oxygen free radical in the human body is the superoxide anion radical (O_2_^•−^) [20,21], which dismutates to form hydrogen peroxide (H_2_O_2_), which can further react to form the hydroxyl radical (HO^•^) [22]. If the production of reactive oxygen species overwhelms the intrinsic anti-oxidant defenses [20], then oxidative stress will arise and this will induce inflammation and endothelial dysfunction [23,24,25,26,27]. It is known that the excess generation of superoxide can directly quench nitric oxide (NO), thereby reducing NO bioavailability and limiting its physiological effects, such as vasodilation [28,29]. 

Although there are some studies on oxidative stress in the blood of patients with CHD, in general they have used indirect or nonspecific markers [30,31,32,33,34,35,36,37,38] or have been based on a certain subgroup of patients [39]. Inflammation, exercise capacity and endothelial function have been studied in CHD, but not their relationship with superoxide anion radical formation.

The aim of the present study was to investigate oxidative stress in adults with CHD. To this end, we determined superoxide anion radical levels as a direct parameter of oxidative stress in the blood of patients with different types of CHD by electron paramagnetic/spin resonance spectroscopy (EPR/ESR) using the spin-probe CMH (hydroxy-3-methoxycarbonyl-2,2,5,5-tetramethylpyrrolidine) [40]. Subsequently, we examined if superoxide anion radical levels were correlated with inflammation, exercise capacity and endothelial dysfunction in adults with CHD.

## 2. Material and Methods

### 2.1. Study Population

Thirty-six adult CHD patients, with an age range of 18–65 years, NYHA class I–II, who visited the out-patient clinic at the Antwerp University Hospital, were prospectively enrolled. The exclusion criteria were: professional endurance athlete, class II and III obesity (BMI > 35 kg/m²), smoking, macrovascular coronary artery disease, diabetes mellitus or presence of systemic disease (e.g., malignancies, acute and chronic inflammatory diseases in the preceding 3 months). For each CHD patient, an age- and gender-matched healthy control was included. The same exclusion criteria were applied. The study was carried out according to the principles of the Declaration of Helsinki and the Research and Ethics committee of the Antwerp University Hospital approved the study protocol (Belgian number: B3002020000298). Written informed consent was obtained from all subjects.

### 2.2. EPR for Superoxide Anion Radical Levels

Fasting venous blood samples were obtained in a heparin tube (BD Vacutainer^®^, Mississauga, ON, Canada). The blood was immediately treated with 1-hydroxy-3-methoxycarbonyl-2,2,5,5-tetramethylpyrrolidine (CMH, Noxygen Science Transfer & Diagnostics, Alsace, Germany). A 1 mM CMH solution was prepared in buffer (Krebs-Hepes buffer (KHB)) containing 25 μM deferroxamine methane-sulfonate salt (DF) chelating agent and 5 μM sodium diethyldithio-carbamate trihydrate (DETC)) at pH 7.4. CMH has been shown to be a suitable spin probe for the quantification of superoxide radical anions in blood [41]. Therefore, 100 µL of blood was added to 100 µL of spin probe CMH. Immediately after mixing, the sample was snap frozen and stored at −80 °C until analysis. For analysis, the mixture of CMH and blood was thawed and transferred into a 50 μL glass capillary (Hirschmann^®^, Eberstadt, Germany). The glass capillaries were placed in the resonator of the EPR. EPR measurements were carried out on a Bruker EMX 1273 spectrometer equipped with an ER 4119HS high-sensitivity resonator and 12 kW power supply operating at X band frequencies [21,42]. The EPR analysis setting were as follows: frequency, 9.86 GHz; power, 50.41 mW; modulation frequency, 100 kHz; modulation amplitude, 1 G; sweep time, 41.94 s; time constant, 40.96 ms; sweep width, 50 G; number of scans, 1 [21]. The data were analyzed using WinEPR (Brüker, Bremen, Germany) software and radicals were identified and quantified as ESR peak amplitude arbitrary units (A.U.). We took two samples from each participant. The intraclass correlation coefficient was 0.921 (one-way random effects, single measures), indicating that there was a good agreement between the two measurements. Therefore, we performed the statistical analysis by using the first sample measured. A Bland–Altman plot and scatter plots are shown in Figure S1 in the Supplementary Materials.

### 2.3. Haematological Parameters

Fasting peripheral blood was collected using ethylenediaminetetraacetic acid (EDTA) and serum vacuette tubes (BD Vacutainer^®^, Mississauga, ON, Canada). EDTA and serum samples were analyzed using a Sysmex XN-9100 (Sysmex, Norderstedt, Germany) and Atellica^®^ IM/CH Analyzer (Siemens Healthcare, Erlangen, Germany), respectively. Blood cell counts, high-sensitivity C-reactive protein (hs-CRP), N-terminal-pro hormone B-type natriuretic peptide (NT-proBNP), fasting glucose, cholesterol levels, iron saturation and folic acid concentrations were quantified.

### 2.4. Physical Activity Level

All participants were asked to complete a physical activity level questionnaire: IPAQ-SF [43].

### 2.5. Exercise Capacity

A cardiopulmonary exercise test (CPET) was performed in the CHD patient group. In brief, a continuously incrementing ramp protocol (an increase in work rate, e.g., every 2–15 s) was designed with the aim of reaching maximal exertion within 8–12 min on a Lode Corival bike ergometer. The increase in load was based on Jones’ predictions of Wattmax [44]. The gas exchange measurements and 12-lead electrocardiogram were recorded continuously. Blood pressure was measured every minute. Peak oxygen consumption (pVO_2_) was determined as the mean VO_2_ peak during the final 30 s of exercise.

### 2.6. Vascular Function Measurements

Blood pressure measurements were taken using an automated blood pressure device (Digital ProBP^TM^ 2000, Welch Allyn, Auburn, NY, USA) in all participants. The peripheral endothelial function at the microvascular level was evaluated only in the CHD patient group using the Endo-PAT2000^®^ (Itamar Medical, software version 3.2.4, Caesarea, Israel), as previously described [21]. The EndoPAT system uses pneumatic finger probes that assess digital volume changes accompanying pulse waves. Relative ischemia was induced by inflating a blood pressure cuff to at least 100 mmHg above systolic blood pressure, on the forearm of the patient for five minutes, after which the pressure was released and reactive hyperemia was measured. Reactive hyperemia induces an increase in shear stress, resulting in an increase in endothelial NO production and subsequent vasodilation. The reactive hyperemia index (RHI) was calculated based on the ratio of the average amplitude of the PAT signal over a one-minute period starting one minute after cuff deflation (maximum pulse amplitude) divided by the average amplitude of the PAT signal over a 3.5 min period before cuff inflation (baseline pulse amplitude). The control arm was used to correct for confounding factors (room temperature, systemic changes). EndoPAT is an operator-independent and highly reproducible technique [45].

### 2.7. Statistical Analysis

Statistical analysis was performed using SPSS version 28.0. The normality of the continuous variables was evaluated using histograms and Q-Q plots. Because some of the parameters were not normally distributed, non-parametric testing was performed. Data are presented as the median (Q1–Q3). Groups were compared using the Mann–Whitney U test and Fisher’s exact test for continuous and categorical variables, respectively. Spearman’s correlation coefficient was used for univariable correlation analysis. Correlations between superoxide on the one hand, and age, BMI, alcohol consumption, blood pressure, thrombocyte and white blood cell count, hs-CRP, NT-proBNP, iron saturation, folic acid, pVO_2_ and RHI on the other, were investigated. A two-tailed *p* < 0.05 was considered significant.

## 3. Results

### 3.1. Characteristics, Haematological Parameters and Self-Reported Physical Activity Levels

The characteristics, hematological parameters and self-reported physical activity levels of both the patient and control group are summarized in Table 1. Patients differed from healthy controls in systolic and diastolic blood pressure, white blood cell count, fasting glucose and NT-proBNP levels.

Of all the patients, 15 (41.7%) had cyanotic and 21 (58.3%) had acyanotic CHD. The different types of CHD in the patient population are shown in Table 2. Nineteen patients (52.8%) had a surgical history for CHD, whereas 17 (42.2%) never had surgery.

### 3.2. Superoxide Anion Radical Levels

**Figure 1:**
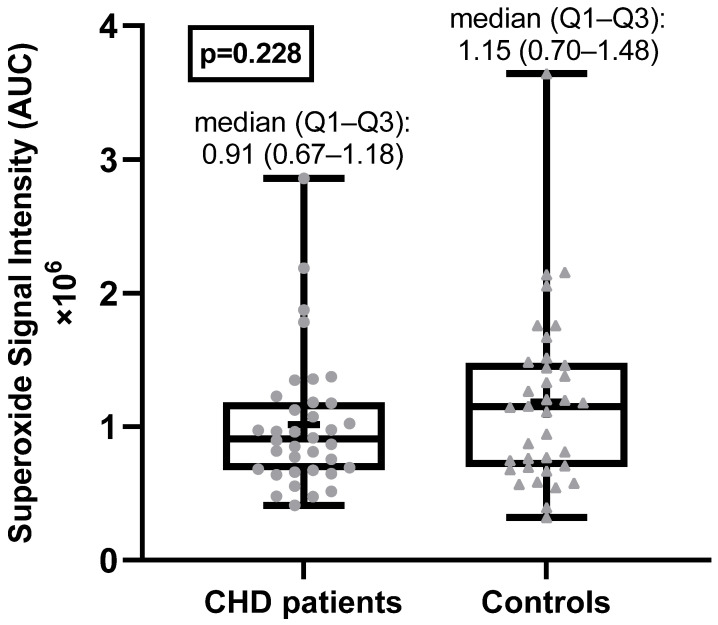
Superoxide anion radical levels in CHD patients and healthy controls. AU = arbitrary units.

**Figure 2:**
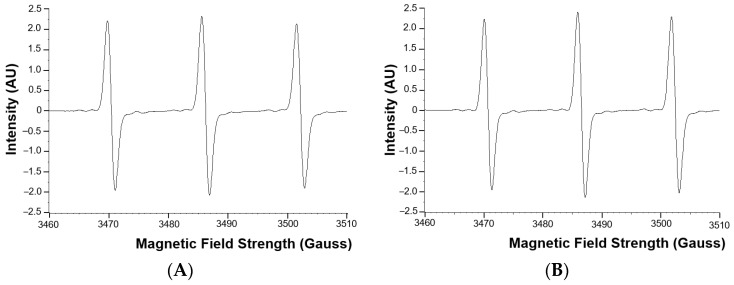
Examples of EPR spectra of the CMH radical detected in whole blood of a typical patient (**A**) and matched healthy control (**B**). AU = arbitrary units.

The superoxide anion radical levels in the blood of adult CHD patients were not statistically significantly different from the superoxide anion radical levels in the healthy control group (Figure 1). Examples of EPR spectra of the CMH radicals detected in whole blood of a typical patient and matched healthy control are shown in Figure 2.

### 3.3. Oxidative Stress and Characteristics and Haematological Parameters

There was a statistically significant positive correlation between superoxide anion radical levels and BMI, but only for the healthy control group (Table 3 and Figure S4 in the Supplementary Materials) and a significant negative correlation between superoxide anion radical levels and alcohol consumption for the healthy control group and the total group (Table 3 and Figure S4 in the Supplementary Materials). There was no significant correlation with age or blood pressure. Also, superoxide anion radical levels were not significantly different between males and females (Figure S2 in the Supplementary Materials). There was no statistically significant difference in superoxide anion radical levels between cyanotic and acyanotic patients (Figure S3A in the Supplementary Materials) nor between patients with and without a surgical history (Figure S3B in the Supplementary Materials).

The superoxide anion radical levels were significantly and positively correlated with the thrombocyte count in healthy controls and in the total group (Table 3 and Figure S5 in the Supplementary Materials). While superoxide anion radical levels significantly and positively correlated with white blood cell count in healthy controls (Table 3 and Figure S5 in the Supplementary Materials), there was no significant correlation between the superoxide anion radical level and hs-CRP in any group. Iron saturation turned out to be significantly negatively correlated with superoxide anion radical levels in patients and in the total group (Table 3 and Figure S6 in the Supplementary Materials) as did folic acid in the total group (Table 3 and Figure S6 in the Supplementary Materials). There were no significant associations between superoxide anion radical levels and NT-proBNP.

### 3.4. Oxidative Stress and Exercise Capacity in Adults with CHD

Only exercise tests that were performed maximally (RER > 1.10) were included in the analysis. The median pVO_2_ in the patient group was 26.45 (21.05–32.10) mL/kg/min. There was no statistically significant correlation between pVO_2_ and superoxide anion radical levels in the blood of CHD patients (Table 3).

### 3.5. Oxidative Stress and Peripheral Microvascular Endothelial Function in Adults with CHD

The median RHI in the patient group was 2.17 (1.78–2.68). There was no statistically significant correlation between the RHI and superoxide anion radical levels in the blood of CHD patients (Table 3).

## 4. Discussion

Oxidative stress is involved in the pathophysiology of endothelial dysfunction and most cardiovascular disorders, but studies in adult patients with CHD are scarce. Therefore, we measured superoxide anion radicals directly with EPR in a heterogeneous population of CHD and studied its relationship with patient characteristics, biochemical parameters, exercise capacity and vascular function tests. The main finding of this study was that the blood levels of superoxide anion radical did not significantly differ between adults with CHD and their age- and gender-matched healthy controls. This finding is contrary to some other studies that claim the presence of oxidative stress in the peripheral blood of patients with CHD [30,31,32,33,34,35,36,37,38,39], but it is in accordance with three studies showing that there was no significant difference in oxidative stress in the peripheral blood of patients with CHD and healthy individuals [46,47,48].

Notably, just one other study [39] measured superoxide anion radical levels in the blood directly with EPR in combination with CMH; superoxide anion radical levels were increased in 18 infants with increased pulmonary blood flow due to ventricular septal defects (VSD). In our study, only five adult patients with VSD were included; this subgroup was too small to perform a separate statistical analysis. In contrast, in most other studies on oxidative stress in the blood of patients with CHD, indirect nonspecific markers of oxidative stress [30,31,32,33,34,35,36,37,38,46,47,48,49,50,51,52,53,54,55,56,57,58,59,60,61,62,63,64,65,66] were used, e.g., the assessment of the activity of oxidative and antioxidant enzymes (superoxide dismutase (SOD), catalase and glutathione peroxidase), levels of antioxidants (vitamin E, uric acid and selenium), levels of malondialdehyde (MDA) and protein carbonyl (PCO), plasma total oxidant status (TOS), total antioxidant capacity or status (TAC or TAS) and oxidative stress index (OSI) as well as the quantification of DNA damage assessed by alkaline comet assay in circulating lymphocytes and levels of 8-hydroxy-2′-deoxyguanosine.

In our adult CHD population, there was no significant difference in superoxide anion radical levels between cyanotic and acyanotic patients, or between patients with and without a surgical history. This is in contrast to two other studies [30,34] showing that the level of oxidative stress in children with cyanotic CHD was significantly higher than in the acyanotic group. One study even examined the difference in oxidative stress between different types of left-to-right shunt CHD. They concluded that the SOD and MDA contents in erythrocytes can be used as markers for the assessment of the severity of the disease [31].

Moreover, oxidative stress was not correlated with inflammation or with endothelial function or exercise capacity in our adult CHD patient group. Although there was a statistically significant correlation between superoxide anion radical levels and white blood cell count in the control group, this was not the case in the patient group. This is in contrast to the results of Pirinccioglu et al. [34] and Michel et al. [32]. The former author group found that blood levels of MDA and PCO were positively correlated with hs-CRP in their group of children with cyanotic and acyanotic CHD. Moreover, PCO was also positively correlated with pro-inflammatory cytokine IL-6 [34]. The latter authors studied alterations in the amino acid metabolome in adult Fontan patients, suggesting links between Fontan pathophysiology, altered cell energy metabolism, oxidative stress and endothelial dysfunction [32]. However, RHI and superoxide anion radical level were not correlated in our patients, thereby not confirming a link between endothelial dysfunction and oxidative stress in CHD. Although the EndoPAT^®^ has been used several times to measure peripheral endothelial function in CHD [67,68,69], it has never been correlated with direct measurements of superoxide anion radicals. In patients with preeclampsia, on the other hand, the association between oxidative stress and microvascular endothelial function has been studied. Mannaerts et al. [21] found a significant relationship between increased superoxide concentration and decreased RHI in women with preeclampsia, but there was no significant correlation between the superoxide and hematological parameters of systemic inflammation (mean platelet volume and neutrophil–lymphocyte ratio). In patients with chronic HF, oxidative stress evaluated by the measurement of free radicals in venous blood using EPR, similar to our study, was also not related to endothelial function [19] in accordance with our study results. However, endothelial function was examined with flow-mediated dilation (FMD) in the brachial artery, which is a conduit artery, and therefore it does not study the microcirculation. Using indirect assessments of oxidative stress, two groups [32,47] have already established a negative association between oxidative stress and exercise capacity in CHD. In our study, we were unable to confirm this finding as there was no statistically significant correlation between pVO_2_ and superoxide anion radical levels directly measured by EPR in the blood of CHD patients.

Iron saturation negatively correlated with oxidative stress in adults with CHD, but not in the control group. It is well-known that a complex interplay exists between iron metabolism and reactive oxygen species (ROS), such as superoxide [70]. Iron is reported to be involved in both the formation and the scavenging of these species [70]. Since iron can be a necessary cofactor in the production of free radicals, iron excess is related to oxidative damage. On the contrary, iron deficiency results in defective mitochondrial function and mitochondrial DNA damage, which results in the release and leakage of ROS out of deficient mitochondria. Therefore, both iron deficiency and iron excess could promote oxidative stress [21,71,72].

Regarding the BMI, there is mounting evidence that human obesity is a state of chronic oxidative stress with increased superoxide production [73]. In children with CHD, BMI was negatively correlated with MDA and PCO while it was positively correlated with TAC [34]. Interestingly, our findings are similar: a statistically significant positive correlation between superoxide and BMI in the control group, and a negative trend—although not significant—in CHD (Table 3). In our control group, the thrombocyte count was positively correlated with superoxide levels. It is known that the oxidative stress and decreased antioxidant levels found in cardiovascular disease are associated with changes in platelet function [74]. Also, the adhesion of activated platelets to the leukocytes greatly enhances the capacity of the leukocyte to generate superoxide [75,76]. Nevertheless, we found no significant correlation between superoxide and the thrombocyte count in our patient group. Finally, there are multiple studies suggesting that folic acid may offer a protective effect against oxidative stress and inflammatory responses and that it has beneficial effects on endothelial function [77,78,79]; however, our findings also seem to suggest that alcohol has a protective effect against oxidative stress. The effects of chronic alcohol exposure on the cellular content or activity of SOD are controversial, with reports of increases, no changes, or decreases, depending on the model, diet, amount, and time of alcohol feeding [80]. In our study population, all but one male healthy control belonged to the no, light or moderate drinker groups (i.e., ≤14 drinks a week for men, ≤7 drinks per week for women). Although several studies have shown a protective effect of moderate alcohol consumption on the incidence of cardiovascular diseases [81,82], other research has shown that alcohol consumption of all amounts is associated with increased cardiovascular risk [83] and that the risk of all-cause mortality, and of cancers specifically, rises with increasing levels of consumption; the level of consumption that minimizes health loss is zero [84]. Therefore, a chronic intake of low to moderate amounts of alcohol cannot be recommended.

The goal of this study was to measure superoxide anion radical levels in the whole blood of adults with CHD, which provides a representation of possible general stress originating from blood cells and the endothelium. These superoxide anion radical levels in whole blood may reflect the contribution of multiple organs, but do not indicate changes in specific tissues or organs. Although there are already indications for oxidative stress in, for example, the myocardial tissue of patients with CHD [85,86], indirect nonspecific biomarkers of oxidative stress were used in these studies. In follow-up studies, it would be interesting to measure superoxide anion radical levels more directly, for example, by EPR, in selected organs or tissues, such as in the heart muscle.

Overall, whole blood superoxide anion radical levels in adults with CHD were not elevated, but iron levels seem to play a more important role in oxidative stress mechanisms in CHD than in healthy controls. It will be worthwhile to investigate these outcomes in studies with a larger sample size on the one hand, and in studies focusing on one specific CHD on the other hand.

## 5. Conclusions

In conclusion, there was no difference in superoxide anion radical levels in whole blood between adult patients with CHD and healthy controls. Moreover, oxidative stress did not correlate with inflammation or with endothelial function or exercise capacity in the patient group; however, a statistically significant negative correlation with iron saturation was found. This is the first paper to directly measure superoxide anion radicals with EPR in a varied population of adults with CHD and to correlate this direct oxidative stress marker with the characteristics, biochemical parameters, exercise capacity and endothelial function in this patient population. More research, especially studies with a large sample size, will be needed to improve our understanding of the underlying pathophysiology of CHD.

## Figures and Tables

**Table 1:** Characteristics, hematological parameters and self-reported physical activity levels of the CHD patients and healthy controls.

Variable	CHD Patients (n = 36)	Controls (n = 36)	*p*-Value
Characteristics			
Age (years)	31.50 (24.25–41.50)	31.50 (24.25–41.50)	1.000
Gender (M/F)	18/18	18/18	1.000
BMI (kg/m^2^)	24.39 (21.73–27.34)	24.33 (21.26–27.77)	0.813
Alcohol consumption (n glasses/week)	1.00 (0.31–2.75)	2.75 (0.63–4.00)	0.052
Systolic blood pressure (mmHg)	114.50 (108.00–121.50)	121.00 (116.75–129.25)	0.004 *
Diastolic blood pressure (mmHg)	72.00 (68.25–76.75)	80.50 (70.75–85.00)	0.001 *
Heart rate at rest (bpm)	63.50 (57.25–73.75)	67.50 (62.25–81.00)	0.114
Hematological parameters			
Hemoglobin (g/dL)	14.70 (13.50–15.70)	14.65 (13.95–15.85)	0.849
Hematocrit (%)	44.10 (40.70–46.90)	44.25 (42.08–47.45)	0.516
Thrombocyte count (×10^9^ E/L)	251.00 (214.00–280.00)	251.50 (204.50–285.50)	0.913
White blood cell count (×10^9^ E/L)	6.38 (5.80–7.32)	5.63 (4.84–6.53)	0.026 *
hs-CRP (mg/L)	0.76 (0.42–2.15)	0.97 (0.40–2.15)	0.608
NT-proBNP (pg/mL)	82.00 (38.50–158.25)	46.50 (35.00–86.00)	0.017 *
Fasting glucose (mg/dL)	86.00 (79.00–89.00)	90.50 (86.25–95.00)	<0.001 *
Total cholesterol (mg/dL)	177.50 (155.25–209.50)	184.50 (162.75–206.75)	0.290
LDL (mg/dL)	128.50 (96.25–151.75)	136.00 (108.50–147.75)	0.551
HDL (mg/dL)	50.50 (43.25–65.75)	54.50 (44.75–69.25)	0.373
Triglycerides (mg/dL)	88.00 (67.25–126.25)	98.50 (76.25–137.50)	0.350
Iron saturation (%)	36.00 (28.00–43.50)	32.00 (23.00–38.00)	0.103
Folic acid (µg/L)	9.20 (7.20–10.90)	9.35 (7.00–11.05)	0.679
Self-reported physical activity level			
Walking MET	594.00 (248.00–2079.00)	643.50 (297.00–1386.00)	0.616
Moderate MET	360.00 (0.00–960.00)	480.00 (0.00–960.00)	0.902
Vigorous MET	480.00 (0.00–1800.00)	720.00 (0.00–1560.00)	0.938
Total MET	2853.00 (822.00–4994.00)	2283.00 (926.25–4536.00)	0.228

Data are presented as the median (Q1–Q3); * The significance level is 0.05; Abbreviations: BMI = body mass index, F = female, HDL = high-density lipoprotein, hs-CRP = high-sensitivity C-reactive protein, LDL = low-density lipoprotein, M = male, MET = metabolic equivalent of task, n = amount, NT = N-terminal-pro hormone B-type natriuretic peptide.

**Table 2:** Occurrence of different types of CHD.

Type of CHD	Number of Patients	Type of CHD	Number of Patients
TGA *	5	ASD II	5
TGA + VSD	1	ASD II + TI	1
TOF	3	ASD II + BAV	1
PS	3	BAV	8
PS + VSD	1	UAV	1
VSD	3	PDA	1
AVSD/ASD + VSD	2	Cor triatriatum sinister	1

* including 1 ccTGA. Abbreviations: ASD = atrial septal defect, AVSD = atrioventricular septal defect, BAV = bicuspid aortic valve, PDA = patent ductus arteriosus, PS = pulmonary valve stenosis, (cc)TGA = (congenitally corrected) transposition of the great arteries, TI = tricuspid insufficiency, TOF = Tetralogy of Fallot, UAV = unicuspid aortic valve, VSD = ventricular septal defect.

**Table 3:** Correlation coefficients between superoxide anion radical levels and characteristics, hematological parameters, exercise capacity and peripheral microvascular endothelial function.

Variable	CHD Patients		Controls		Total Group	
	r	*p*-Value	r	*p*-Value	r	*p*-Value
Characteristics						
Age (years)	−0.239	0.161	0.001	0.996	−0.116	0.332
BMI (kg/m^2^)	−0.170	0.321	**0.451**	0.006 **	0.172	0.148
Alcohol consumption (n glasses/week)	−0.237	0.164	**−0.330**	0.050 *	**−0.254**	0.032 *
Systolic blood pressure (mmHg)	−0.176	0.305	0.214	0.224	0.041	0.738
Diastolic blood pressure (mmHg)	−0.303	0.072	0.073	0.680	−0.078	0.520
Haematological parameters						
Thrombocyte count (×10^9^ E/L)	0.163	0.350	**0.436**	0.008 **	**0.285**	0.016 *
White blood cell count (×10^9^ E/L)	−0.068	0.699	**0.461**	0.005 **	0.194	0.105
hs-CRP (mg/L)	−0.015	0.929	0.249	0.143	0.172	0.148
NT-proBNP (pg/mL)	0.094	0.585	0.292	0.084	0.170	0.153
Iron saturation (%)	**−0.367**	0.028 *	−0.196	0.251	**−0.274**	0.020 *
Folic acid (µg/L)	−0.290	0.092	−0.265	0.118	**−0.288**	0.015 *
Exercise capacity						
pVO_2_ (ml/kg/min)	0.332	0.084	/		/	
Peripheral microvascular endothelial function						
RHI	0.177	0.301	/		/	

BMI = body mass index, hs-CRP = high-sensitivity C-reactive protein, n = amount, NT = N-terminal-pro hormone B-type natriuretic peptide, pVO_2_ (ml/kg/min) = peak oxygen consumption, RHI = reactive hyperemia index. r = Spearman’s rho. * Correlation is significant at the 0.05 level (2-tailed), ** Correlation is significant at the 0.01 level (2-tailed).

## Data Availability

The data presented in this study are available on request from the corresponding author. The data are not publicly available due to privacy and ethical restrictions.

## Supplementary Materials

The following supporting information can be downloaded at: https://www.mdpi.com/article/10.3390/antiox11122369/s1, Figure S1: Bland-Altman visualisation (A) and scatter plot (B) of the two samples measuring superoxide anion radical levels. Figure S2: Comparison of superoxide anion radical levels between male and female subjects in CHD patients (A), healthy controls (B) and the total group (C). Figure S3: Comparison of superoxide anion radical levels between cyanotic and acyanotic CHD (A) and CHD patients with and without a surgical history (B). Figure S4: Correlation of superoxide anion radical levels with BMI and alcohol consumption in CHD patients (A + D), healthy controls (B + E) and the total group (C + F). Figure S5: Correlation of superoxide anion radical levels with thrombocyte count and white blood cell count in CHD patients (A + D), healthy controls (B + E) and the total group (C + F). Figure S6: Correlation of superoxide anion radical levels with iron saturation and folic acid in CHD patients (A + D), healthy controls (B + E) and the total group (C + F).

## Abbreviations

BMI	body mass index
CHD	congenital heart disease
CMH	hydroxy-3-methoxycarbonyl-2,2,5,5-tetramethylpyrrolidine
CPET	cardiopulmonary exercise testing
EDTA	ethylenediaminetetraacetic acid
EPR/ESR	electron paramagnetic/spin resonance spectroscopy
FMD	flow-mediated dilation
H2O2	hydrogen peroxide
HF	heart failure
HO•	hydroxyl radical
hs-CRP	high-sensitivity C-reactive protein
IPAQ-SF	International Physical Activity Questionnaire-Short Form
MDA	Malondialdehyde
NT-proBNP	N-terminal-pro hormone B-type natriuretic peptide
NYHA	New York Heart Association
pVO_2_	peak oxygen consumption
O2•^−^	superoxide anion radical
OSI	oxidative stress index
PCO	protein carbonyl
RER	respiratory exchange ratio
RHI	reactive hyperemia index
ROS	reactive oxygen species
SOD	superoxide dismutase
TAC/TAS	total antioxidant capacity/status
TOS	total oxidant status
VSD	ventricular septal defect
