# Supplementary material for: Circulating Reactive Oxygen Species in Adults with Congenital Heart Disease
Source: Antioxidants (Basel). 2022 Nov 30;11(12):2369. doi: 10.3390/antiox11122369 (PMC9774177; doi:10.3390/antiox11122369)
Supplement: Supplementary file 1 [file antioxidants-11-02369-s001.zip › antioxidants-2032922-supplementary.pdf]

Figures

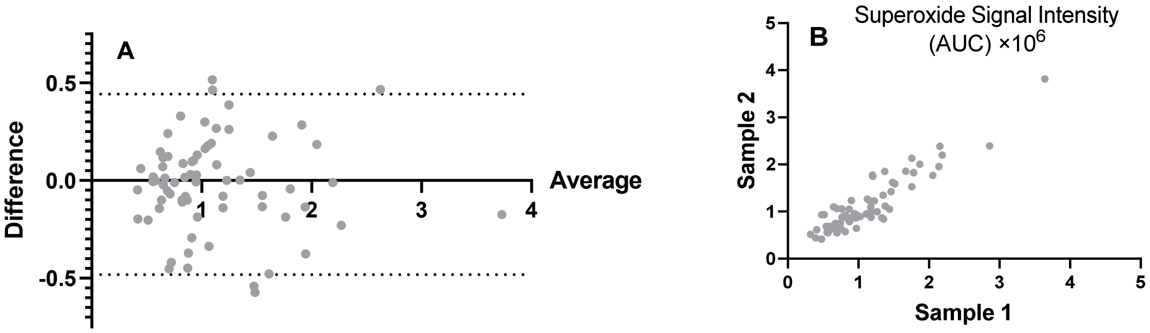

Figure S1. Bland-Altman visualisation (A) and scatter plot (B) of the two samples measuring superoxide anion radical levels. Limits of agreement: lower -0.48, upper 0.44.

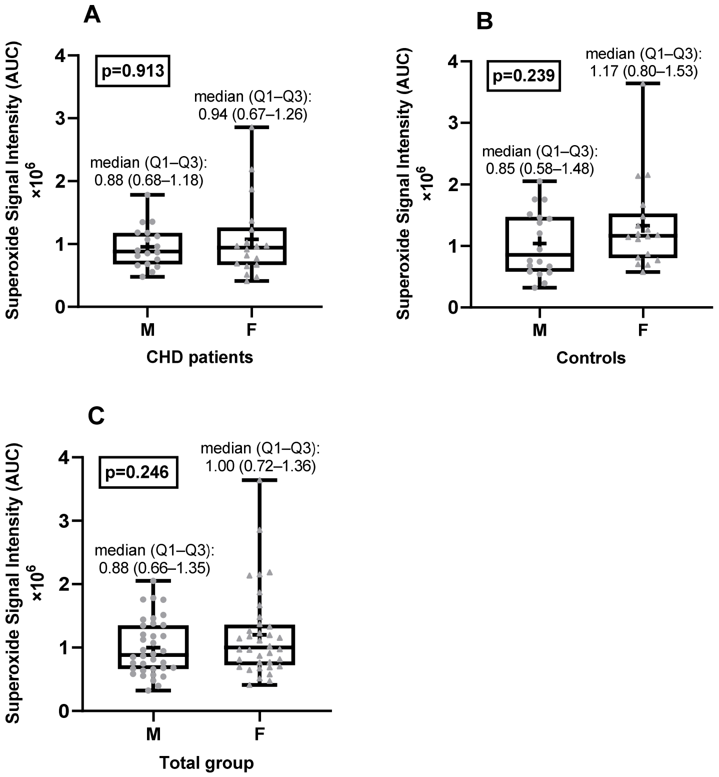

Figure S2: Comparison of superoxide anion radical levels between male and female subjects in CHD patients (A), healthy controls (B) and the total group (C). AUC = area under the curve, M = male, F = female.

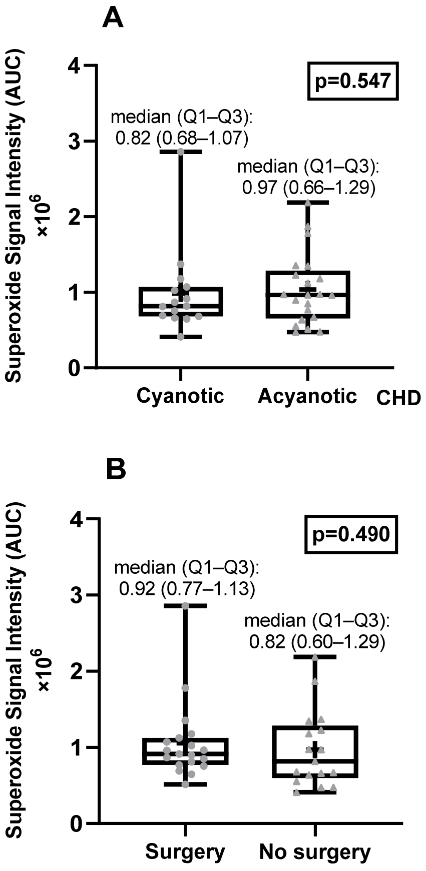

Figure S3. Comparison of superoxide anion radical levels between cyanotic and acyanotic CHD (A) and CHD patients with and without a surgical history (B). AUC = area under the curve, CHD = congenital heart disease

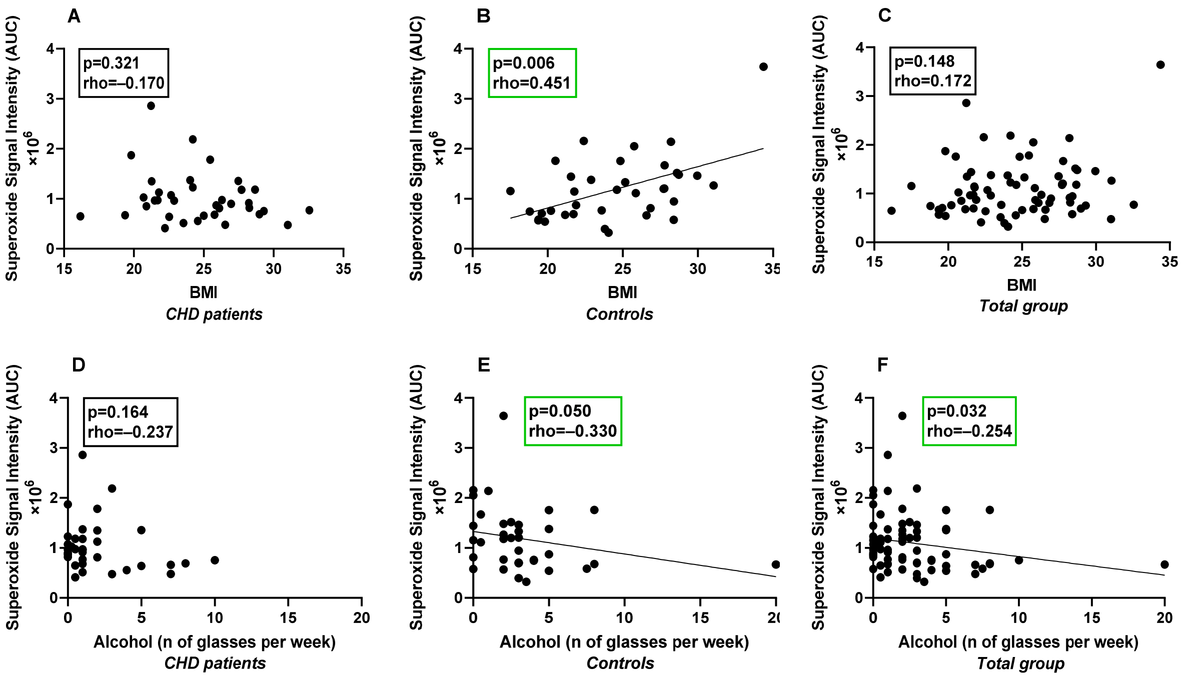

Figure S4. Correlation of superoxide anion radical levels with BMI and alcohol consumption in CHD patients (A+D), healthy controls (B+E) and the total group (C+F). AUC = area under the curve, BMI = body mass index, n = amount, rho = Spearman's rho.

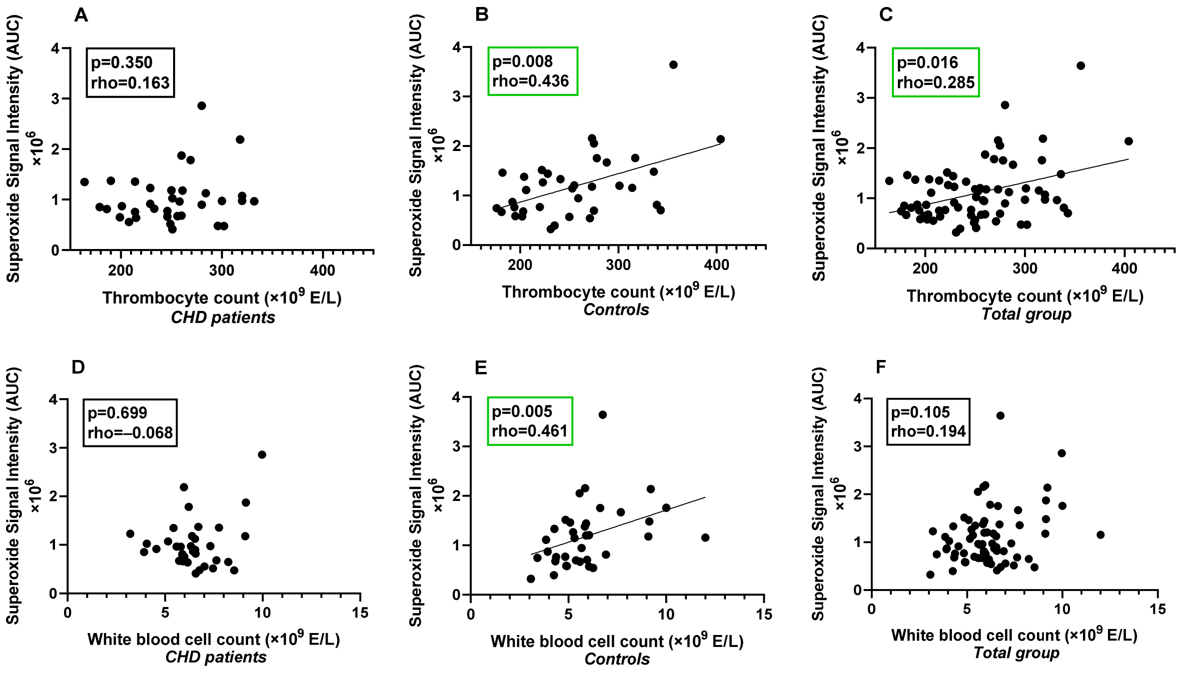

Figure S5. Correlation of superoxide anion radical levels with thrombocyte count and white blood cell count in CHD patients (A+D), healthy controls (B+E) and the total group (C+F). AUC = area under the curve, rho = Spearman's rho.

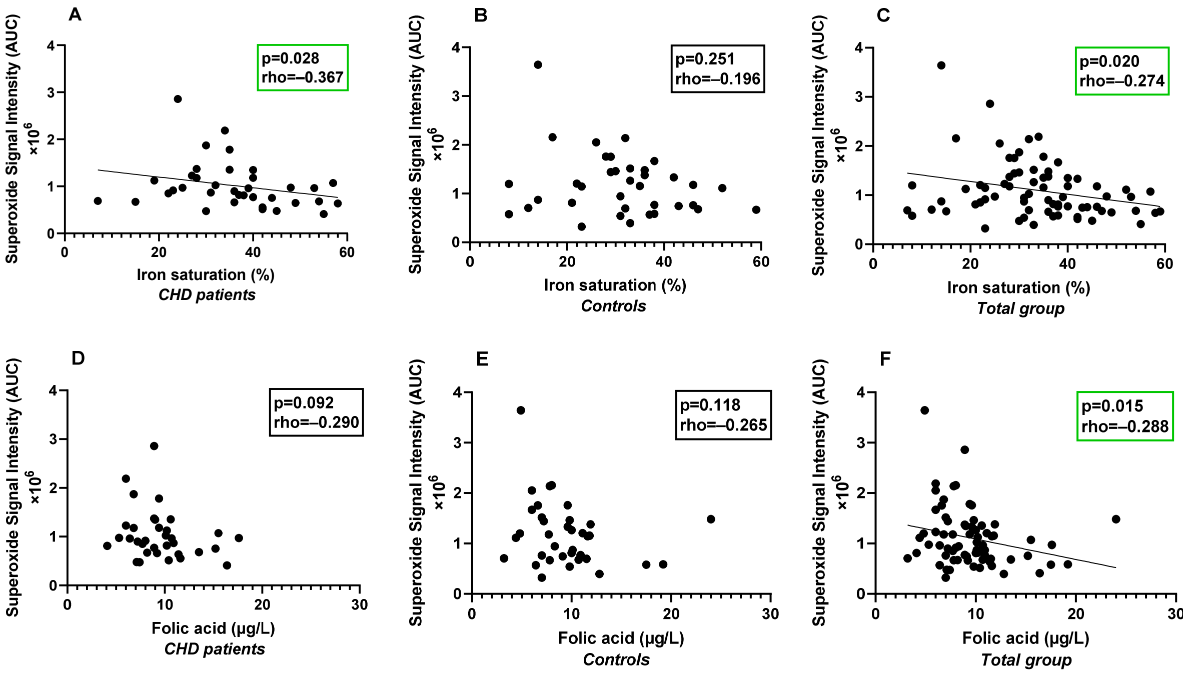

Figure S6. Correlation of superoxide anion radical levels with iron saturation and folic acid in CHD patients (A+D), healthy controls (B+E) and the total group (C+F). AUC = area under the curve, rho = Spearman's rho.
